# Loss of Circulating CD4 T Cells with B Cell Helper Function during Chronic HIV Infection

**DOI:** 10.1371/journal.ppat.1003853

**Published:** 2014-01-30

**Authors:** Kristin L. Boswell, Robert Paris, Eli Boritz, David Ambrozak, Takuya Yamamoto, Sam Darko, Kaska Wloka, Adam Wheatley, Sandeep Narpala, Adrian McDermott, Mario Roederer, Richard Haubrich, Mark Connors, Julie Ake, Daniel C. Douek, Jerome Kim, Constantinos Petrovas, Richard A. Koup

**Affiliations:** 1 Immunology Laboratory, Vaccine Research Center, NIAID, NIH, Bethesda, Maryland, United States of America; 2 United States Military HIV Research Program, Rockville, Maryland, United States of America; 3 Human Immunology Section, Vaccine Research Center, NIAID, NIH, Bethesda, Maryland, United States of America; 4 Immunology Core Section, Vaccine Research Center, NIAID, NIH, Bethesda, Maryland, United States of America; 5 ImmunoTechnology Section, Vaccine Research Center, National Institutes of Health, Bethesda, Maryland, United States of America; 6 Division of Infectious Diseases, Antiviral Research Center, University of California San Diego, San Diego, California, United States of America; 7 HIV-Specific Immunity Section, Laboratory of Immunoregulation, NIAID, NIH, Bethesda, Maryland, United States of America; Emory University, United States of America

## Abstract

The interaction between follicular T helper cells (T_FH_) and B cells in the lymph nodes and spleen has a major impact on the development of antigen-specific B cell responses during infection or vaccination. Recent studies described a functional equivalent of these cells among circulating CD4 T cells, referred to as peripheral T_FH_ cells. Here, we characterize the phenotype and in vitro B cell helper activity of peripheral T_FH_ populations, as well as the effect of HIV infection on these populations. In co-culture experiments we confirmed CXCR5+ cells from HIV-uninfected donors provide help to B cells and more specifically, we identified a CCR7^high^CXCR5^high^CCR6^high^PD-1^high^ CD4 T cell population that secretes IL-21 and enhances isotype-switched immunoglobulin production. This population is significantly decreased in treatment-naïve, HIV-infected individuals and can be recovered after anti-retroviral therapy. We found impaired immunoglobulin production in co-cultures from HIV-infected individuals and found no correlation between the frequency of peripheral T_FH_ cells and memory B cells, or with neutralization activity in untreated HIV infection in our cohort. Furthermore, we found that within the peripheral T_FH_ population, the expression level of T_FH_-associated genes more closely resembles a memory, non-T_FH_ population, as opposed to a T_FH_ population. Overall, our data identify a heterogeneous population of circulating CD4 T cells that provides *in vitro* help to B cells, and challenges the origin of these cells as memory T_FH_ cells.

## Author Summary

Follicular T helper cells (T_FH_) interact with B cells within germinal centers of lymphoid tissue to promote the survival, isotype switching and generation of high affinity memory B cells and plasma cells. Recently, a population of circulating CD4 T cells that shares phenotypic and functional characteristics with T_FH_ cells, named peripheral T_FH_ cells, has been identified. The relationship between peripheral T_FH_ cells in the blood and T_FH_ cells within the lymphoid tissue remains unclear, and whether or not peripheral T_FH_ cells can provide insight into T cell and B cell dynamics within lymphoid tissue during infection or vaccination is not understood. Here we characterize peripheral T_FH_ cells and show that unlike T_FH_ cells, peripheral T_FH_ cells secrete a diverse array of cytokines and decrease, rather than increase, during chronic HIV infection. Furthermore, we did not observe a relationship between peripheral T_FH_ cells and memory B cells, or with the production of neutralizing antibodies to HIV. Overall, our data indicate that while peripheral T_FH_ cells share some characteristics with T_FH_ cells, they may not represent a good surrogate to study T cell and B cell dynamics within lymphoid tissue.

## Introduction

Follicular helper CD4 T cells (T_FH_) are crucial for the development of antigen-specific B cells within germinal centers (GC). T_FH_ cells interact through co-stimulatory receptors and provide essential soluble factors (i.e. IL-4, IL-21) to promote the survival, isotype switching and selection of high affinity memory B cells [1]. Phenotypic and gene signature analysis has revealed a highly conserved molecular profile of T_FH_ cells in humans, non-human primates (NHP) and mice, which is characterized by increased expression of Bcl-6, CXCR5, PD-1, ICOS and decreased expression of CCR7 [2]–[4]. Human T_FH_ cells exhibit a polarized cytokine profile characterized by compromised production of T_H1_ cytokines and increased secretion of IL-4, IL-10 and IL-21 [5]. Although IL-21 is characterized as a “hallmark” cytokine of T_FH_ cells, other T_Helper_ subsets produce this cytokine [6].

The origin and differentiation of T_FH_ is unclear, as previous studies found T_FH_ cells can derive from T_H1_ or T_H2_ cells, or independently of other CD4 lineages [7]–[9]. However, it is well established that the transcription factor Bcl-6 regulates several molecules involved in T_FH_ development (i.e. PD-1, IL-21R, CXCR5) [10], [11]. Similarly, the fate of T_FH_, particularly those in the germinal center (GC-T_FH_), following the effector phase of the immune response is unclear. We have recently shown that NHP GC-T_FH_ display compromised *in vivo* cell cycling and are prone to *in vitro* cell death [4]. Other studies have shown that T_FH_ can form a memory pool found in anatomical sites outside the lymph nodes [12]. Hence, T_FH_ cells may adopt a “central memory” phenotype or undergo cell death after the effector phase [13]. In humans, a circulating CD4 T cell population characterized by high CXCR5 expression can provide *in vitro* help for B cell isotype switching and shares functional characteristics with T_FH_ cells [14]. It was proposed that these circulating cells, termed “peripheral T_FH_” (pT_FH_) could represent the memory counterparts of T_FH_ outside the lymphoid organs. Further investigation is needed to establish a direct relationship between T_FH_ cells and pT_FH_ cells.

It is becoming increasingly important to understand the interplay between CD4 T cells and B cells during HIV infection, specifically with relation to the generation of broadly neutralizing antibodies. Chronic HIV/SIV infection results in profound changes in CD4 T cell dynamics in lymph nodes characterized by T_FH_ accumulation and increased ability of non-T_FH_ to egress the lymph node [4], [15]. How this impacts upon the dynamics of pT_FH_ is unknown. Elucidating the biology and dynamics of pT_FH_, and their ability to provide B cell help may be important for our understanding of T_FH_ memory formation during chronic infection, as well as the establishment of immune correlates reflecting the interactions between CD4 T cells and B cells within secondary lymphoid organs. This is of particular interest for monitoring clinical studies where the B cell arm of the immune system is under investigation [16].

Here we define, detect, quantify and characterize peripheral CD4 T cell populations that support B cell differentiation. We show that particular circulating CD4 T cell populations with distinct cytokine profiles have the capacity to help B cells *in vitro*. We further show that the frequencies of pT_FH_ populations are significantly compromised during chronic HIV infection but can recover with antiretroviral treatment (ART), although *in vitro* immunoglobulin production from HIV-infected subjects both on and off ART is reduced compared to healthy subjects. Furthermore, gene expression analysis of pT_FH_ cells and CD4 T cells in tonsil tissue suggest pT_FH_ cells are most closely related to a non-T_FH_ memory population within secondary lymphoid organs. Overall, our data challenge the relationship between pT_FH_ cells and T_FH_ memory cells.

## Results

### Characterization of peripheral T follicular helper (pT_FH_) cells

**Figure 1:**
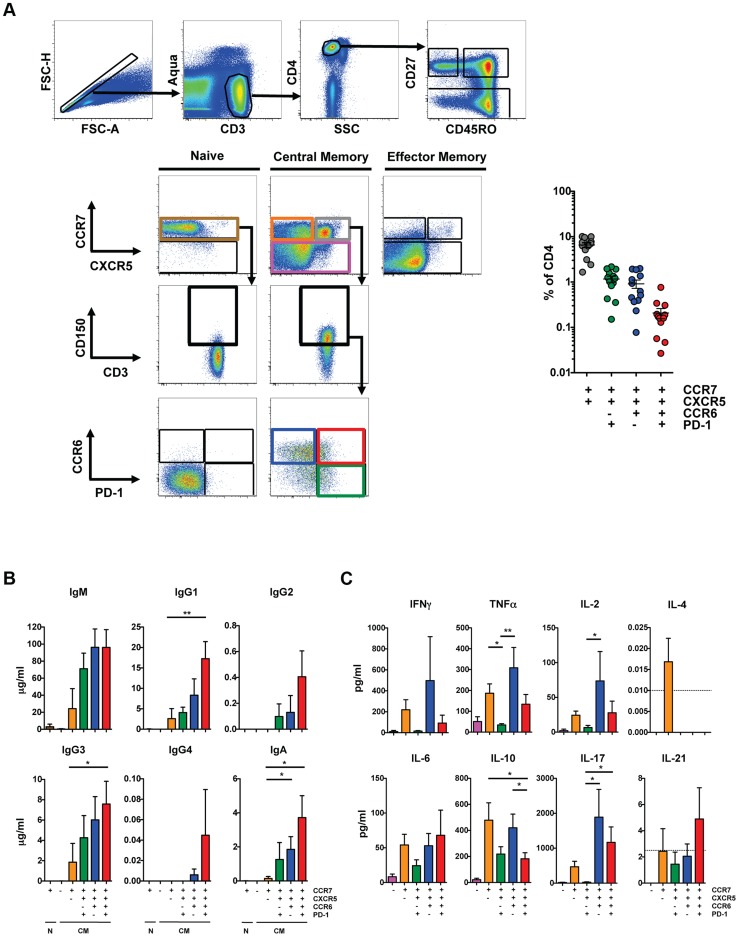
Characterization of peripheral T_FH_ cells. (**A**) Left: Representative flow cytometry plots from HIV-uninfected PBMC showing the gating scheme for isolating T cell subsets for the T cell/B cell coculture assay. Isolated populations include naïve cells (brown), CM CCR7^low^ (pink), CM CCR7^high^CXCR5^low^ (orange), CM CCR7^high^CXCR5^high^CCR6^low^PD-1^high^ (green), CM CCR7^high^CXCR5^high^CCR6^high^PD-1^low^ (blue) and CCR7^high^CXCR5^high^CCR6^high^PD-1^high^ (red). Before gating on CCR6 and PD-1, cells were first gated on CD150^high^. Right: Scatter plot indicating the frequency of each population in HIV-uninfected subjects (*n* = 13). Cells were not gated on CD150 for phenotypic analysis. (**B**) Indicated CD4 T cell populations were cultured with autologous naïve B cells (CD19^high^CD27^low^IgD^−^) in the presence of SEB for 12 days and Ig concentrations were measured from supernatants (n = 6). (**C**) Indicated CD4 T cell populations were cultured with autologous naïve B cells in the presence of SEB for 2 days and cytokine concentrations were measured from supernatants (n = 6). Horizontal lines indicate limit of detection. Significant differences were determined using the Friedman test with Dunn's multiple comparison post-test. *p<0.05; **p<0.01.

Previous studies defined a population of circulating CD4 T cells that express CXCR5, promote the differentiation of naïve B cells and induce immunoglobulin secretion *in vitro* [14], [17]. We further defined CXCR5^high^ CD4 T cells from blood, analyzed their cytokine production and determined their ability to promote B cell differentiation *in vitro*. CXCR5^high^ CD4 T cells were found predominantly within the CD27^high^CD45RO^high^ CD4 T cell population (hereafter referred to as central memory (CM)), in agreement with previous studies [17]. The majority of the CXCR5^high^ CD4 T cell population also expressed CCR7 and we found the CCR7^high^CXCR5^high^ population represented 6.5+/−2.8% (mean+/−S.D.) of total CD4 T cells in healthy subjects (Figure 1A). The majority of CXCR5^high^ cells expressed CD150. We further analyzed these cells based on expression of CCR6, which was previously used in combination with CXCR3 to define a pT_FH_ subset that promotes IgG and IgA production [14], and PD-1. CCR7^high^CXCR5^high^CCR6^high^ cells represented 1.2+/−0.9% of total CD4 T cells and a minority of these cells were PD-1^high^.

To analyze the ability of these populations to promote B cell differentiation, naïve and CM CD4 T cells from HIV-uninfected individuals were sorted based on expression of CCR7, CXCR5, CD150, CCR6 and PD-1 (Figure 1A), and cultured with autologous naïve B cells (CD19^+^CD27^−^IgD^+^) as previously described [14], [18] in the presence of staphylococcal enterotoxin B (SEB). Notably, our sorted naïve B cell population did not express isotype-switched immunoglobulin (Figure S1A) and culture conditions that lacked SEB did not induce immunoglobulin production (data not shown). Naïve and CM CCR7^low^ CD4 T cells failed to promote B cell differentiation and immunoglobulin production whereas CM CCR7^high^CXCR5^low^ cells induced limited production of IgM, IgG1 and IgG3 compared to the CCR7^high^CXCR5^high^ populations (Figure 1B). The CCR7^high^CXCR5^high^CCR6^high^PD-1^high^ population induced the greatest production of IgG1, IgG3 and IgA compared to the CXCR5^low^ population. Prior studies defined pT_FH_ cells based on surface expression of CXCR5, CCR6 and the lack of CXCR3 expression [14]. We found that the greatest help for immunoglobulin production was from CXCR5^high^CCR6^high^ cell populations and, within those, from the PD-1^high^ cells. We did not eliminate a small population of CXCR3^+^ cells in order to avoid removing a larger population of CXCR5^high^CCR6^high^ cells that induce B cell differentiation (Figure S1B).

The cytokine profile of pT_FH_ populations shared characteristics with other T_helper_ subsets, including T_H1_, T_H17_ and T_reg_ cells. Supernatant from the CXCR5^high^CCR6^high^PD-1^low^ coculture contained the greatest quantities of TNF-α, IL-2, and IL-17 compared to the CXCR5^high^CCR6^low^PD-1^high^ coculture (Figure 1C). Notably, the CXCR5^high^CCR6^high^PD-1^high^ population, which promoted the greatest production of IgG1, IgG3 and IgA, showed the greatest IL-21 production, although at low levels.

Overall, CXCR5^high^ CD4 T cell populations induced B cell immunoglobulin production, although the CXCR5^high^CCR6^high^PD-1^high^ population did so most efficiently. However, this population is not characteristic of a T_FH_ population found in secondary lymphoid organs, as coculture supernatants included a broad array of cytokines characteristic of T_FH_ cells and multiple other T_helper_ subsets.

### Progressive loss of pT_FH_ cells in HIV infection

**Figure 2:**
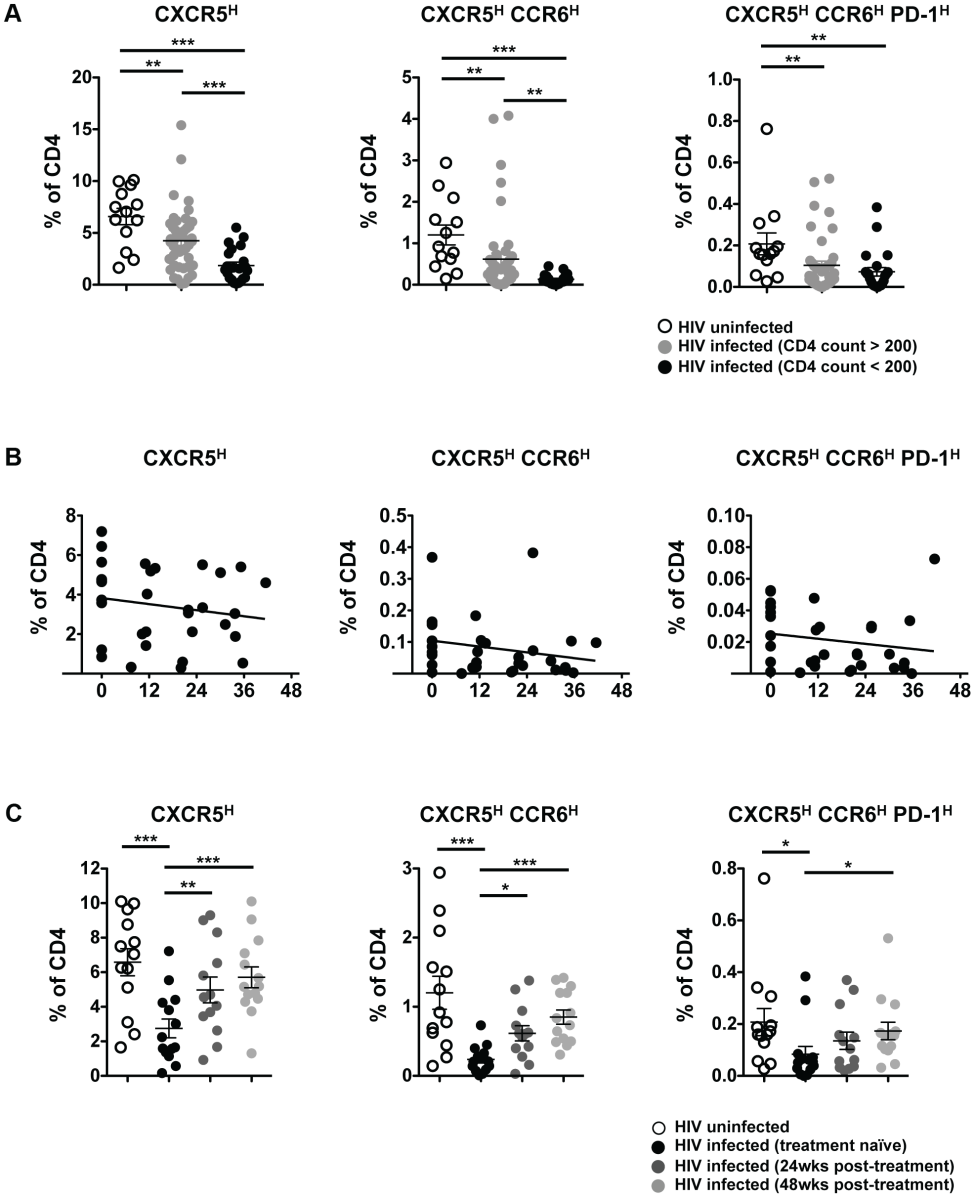
Progressive loss of pT_FH_ cells in HIV infection. (**A**) Pooled data showing the frequency (%) of CXCR5^high^, CXCR5^high^CCR6^high^ and CXCR5^high^CCR6^high^PD-1^high^ populations in total CD4 cells from PBMC from HIV uninfected (open circles; n = 13), HIV-infected (treatment-naïve), CD4 count >200 (light gray circles; n = 44), and HIV-infected (treatment-naïve), CD4 count <200 (black circles; n = 22). Significant differences between HIV-uninfected and HIV-infected subjects were determined using the Mann-Whitney U test. ***p<0.001; **p<0.01; *p<0.05. (**B**) Longitudinal analysis showing the frequency (%) of CXCR5^high^, CXCR5^high^CCR6^high^ and CXCR5^high^CCR6^high^PD-1^high^ populations in total CD4 cells or indicated populations in CXCR5-expressing cells (bottom row) from HIV-infected (treatment naïve) subjects (n = 10) over 36–48 months. No significant correlations were found. (**C**) Pooled data showing the frequency (%) of CXCR5^high^, CXCR5^high^CCR6^high^ and CXCR5^high^CCR6^high^PD-1^high^ populations in total CD4 cells from PBMC from HIV-uninfected subjects (open circles; n = 13) and HIV-infected subjects before (n = 14, week 0; black circles) and after ART (week 24, dark gray circles; week 48, light gray circles). Significant differences between HIV-uninfected and HIV-infected subjects were determined using the Mann-Whitney U test. Significant differences between subjects before and after ART were determined using the Wilcoxon matched-pairs signed rank test. ***p<0.001; **p<0.01; *p<0.05.

To determine the impact of HIV on pT_FH_ populations, we compared pT_FH_ cells from HIV-uninfected subjects and treatment-naïve HIV-infected subjects (Table S1) as a frequency of total CD4 cells. Irrespective of how pT_FH_ cells were defined, there was a significant decrease in the pT_FH_ population from HIV-infected subjects compared to HIV-uninfected subjects (Figure 2A). Subjects with CD4 counts greater than 200 had significantly lower pT_FH_ populations, while subjects with CD4 counts less than 200 had the lowest frequency of all phenotypically defined pT_FH_ populations. However, when we defined the CCR6^high^PD-1^high^ population as a subset of the CXCR5^high^ population, the frequency of the CCR6^high^PD-1^high^ population increased in subjects with CD4 counts less than 200 (Figure S2A). The increase in PD-1^high^ cells was likely due to immune activation in HIV infection, as we observed increases in the frequency of both PD-1^high^ and ICOS^high^ cells within the CXCR5^high^ population, with the greatest increases seen in samples with CD4 counts less than 200 (Figure S2A). We also observed a positive trend between CXCR5^high^PD-1^high^ cells and serum concentrations of soluble CD14. (Figure S2A). For 10 HIV-infected individuals on whom we had longitudinal samples, we observed a loss of pT_FH_ populations as a frequency of total CD4 T cells over 36 to 48 months (Figure 2B). However, the frequency of PD-1^high^, ICOS^high^ and CCR6^high^PD-1^high^ cells within the CXCR5^high^ population remained stable (Figure S2B).

Next, we investigated the impact of ART on the frequency of pT_FH_ within total CD4 T cells. Longitudinal analysis on samples from before and after 24 and 48 weeks of ART revealed a recovery of pT_FH_ populations (Figure 2C). However, the frequency of PD-1^high^, ICOS^high^ and CCR6^high^PD-1^high^ cells remained stable within the CXCR5^high^ population (Figure S2C). Overall, HIV infection causes a loss of pT_FH_ cells and ART promotes the recovery of these populations.

### Impaired B cell help by pT_FH_ cells in HIV infection

**Figure 3:**
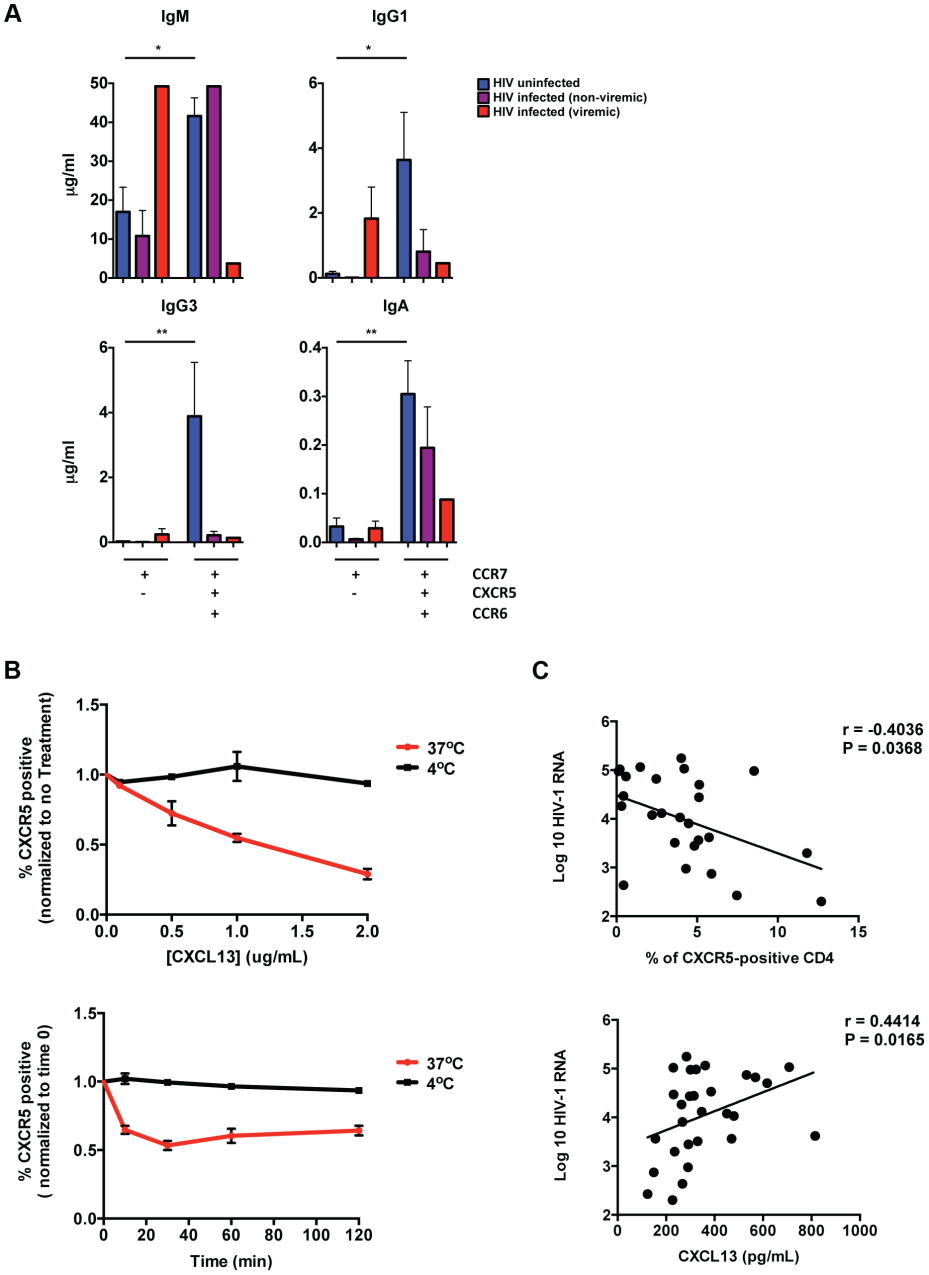
Impaired B cell help by pT_FH_ cells in HIV infection. (**A**) CCR7^high^CXCR5^low^ and CCR7^high^CXCR5^high^CCR6^high^ CM CD4 T cells isolated from PBMCs were cultured with autologous naïve B cells (CD19^high^CD27^low^IgD^−^) in the presence of SEB for 12 days and Ig concentrations were measured from supernatants (HIV-uninfected, n = 8; HIV-infected (non-viremic), n = 5–7, HIV-infected (viremic), n = 1–2). Significant differences were determined using the Wilcoxon paired t-test or the Mann-Whitney test. *p<0.05; **p<0.01. (**B**) Top: HIV-uninfected PBMCs were incubated with indicated concentrations of CXCL-13 for 1 hour at 37°C (red) or 4°C (black). Bottom: Healthy PBMCs were incubated with 1 µg/mL CXCL13 for 10, 30, 60 or 120 minutes at 37°C (red) or 4°C (black). The frequency of CXCR5-positve CD4 T cells was calculated and normalized to time 0. (n = 3). (**C**) Top: Correlative analysis showing the frequency of CM CXCR5-positive CD4 T cells versus viral load (n = 27; r = −0.4036, P = 0.0368). Bottom: Correlative analysis showing the concentration of CXCL-13 in plasma or sera versus viral load (n = 27; r = 0.4414, P = 0.0165). Correlations were analyzed using the nonparametric Spearman test.

To investigate the impact of HIV on the ability of pT_FH_ cells to support B cell differentiation, we performed co-culture experiments with pT_FH_ cells from HIV-infected subjects. We focused on the CXCR5^high^CCR6^high^ population that included both PD-1^high^ and PD-1^low^ cells due to limited cell numbers in HIV-infected subjects. Similar to previous results, the CXCR5^high^CCR6^high^ population from HIV-uninfected subjects supported significantly more immunoglobulin production compared to the CXCR5^low^ population. (Figure 3A). However, for HIV-infected subjects we observed less overall immunoglobulin production when CXCR5^high^CCR6^high^ CD4 T cells were co-cultivated with naïve B cells. Furthermore, in viremic subjects, we observed increased IgM and IgG1 production in co-culture supernatants from the CXCR5^low^ population, compared to HIV-uninfected subjects. Similar to HIV-uninfected subjects, we found that pT_FH_ cells from HIV-infected subjects produced a broad spectrum of cytokines (Figure S3A).

Our data raise the possibility that some pT_FH_ cells exhibit a CXCR5^low^ phenotype in HIV infection. This phenotype could be due the down regulation of CXCR5 on pT_FH_ cells, or indicate the existence of a unique CXCR5^low^ pT_FH_ population in chronic HIV infection. In order to distinguish these two possibilities, we investigated whether CXCL-13 impacts CXCR5 expression on CD4 T cells. We found that incubation of HIV-uninfected PBMC with CXCL-13 led to a decrease in frequency of CXCR5-positive CD4 T cells, presumably due to the internalization of CXCR5 (Figure 3B). Furthermore, in HIV infection we found that viral load positively correlated with CXCL-13 levels and negatively correlated with the frequency of CXCR5-positive CD4 T cells (Figure 3C). However, we did not observe a direct correlation between CXCL13 levels and the frequency of CXCR5-positive CD4 T cells. Importantly, we also found that *in vitro* infection of CXCR5-expressing CD4 T cells did not impact CXCR5 surface expression (Figure S3B). Therefore, our data support the possibility that in untreated HIV infected individuals, increased levels of CXCL-13 could effect CXCR5 surface expression on pT_FH_ cells.

### Defective cytokine production of pT_FH_ in HIV infection

**Figure 4:**
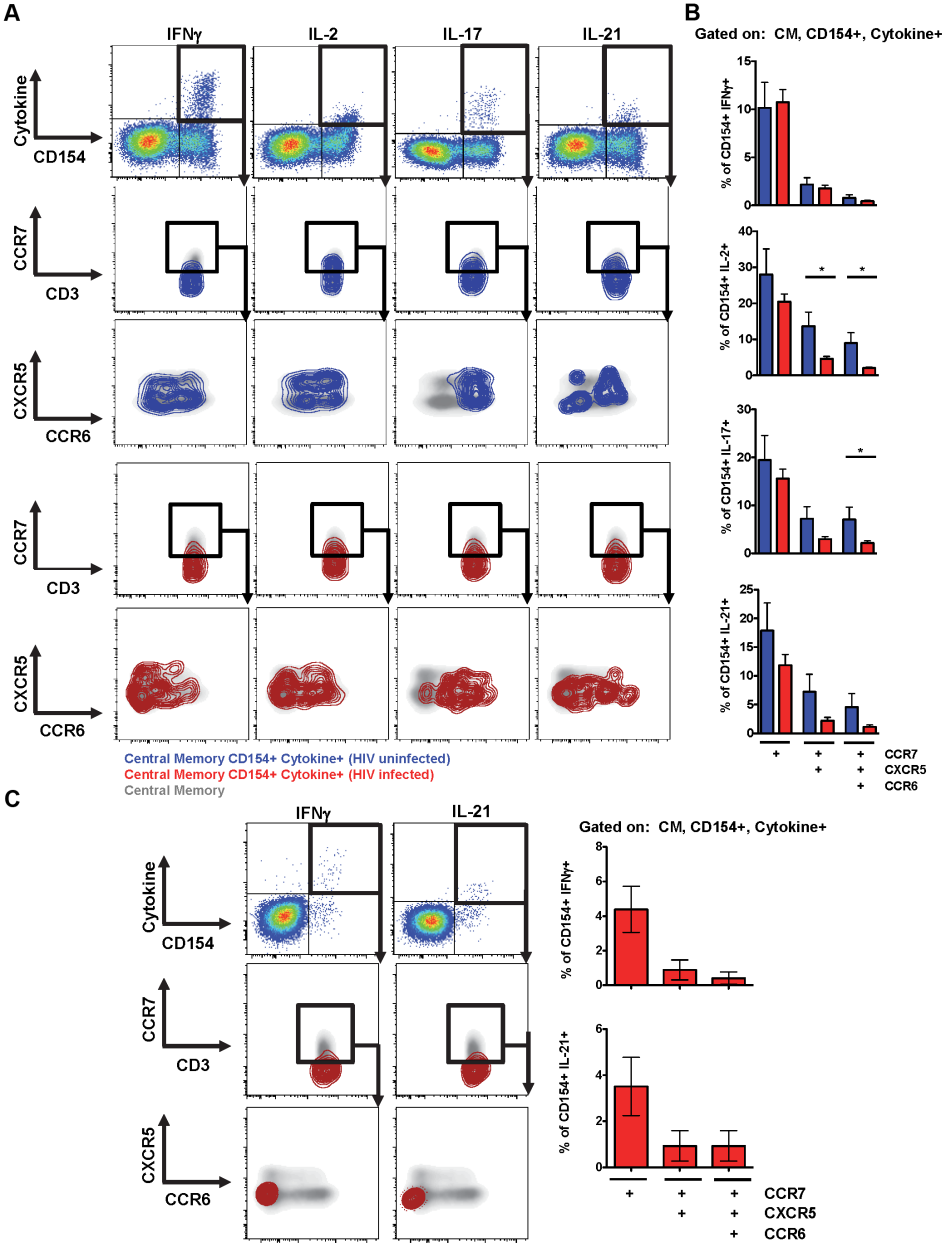
Functional characteristics of pT_FH_ cells and the impact of HIV. (**A**) Representative flow cytometry plots showing CM, CD154-positive, cytokine-positive cells after SEB stimulation. CD154-positive, cytokine-positive CD4 T cells, shown by contour plots (blue: HIV-uninfected; red: HIV-infected), are overlaid onto 2 dimensional density plots for CM CD4 T cells plotted against CCR7 and CD3, and CXCR5 and CCR6. (**B**) Bar graphs showing the frequency of SEB-stimulated CD154-positive, cytokine-positive cells that express CCR7, CXCR5 and CCR6 (Blue: uninfected; n = 5; Red: HIV-infected; n = 24). (**C**) Left: Gag-specific CD4+ T cells (CD154-positive, cytokine-positive) shown as red contour plots are overlaid onto 2 dimensional density plots for CM cells CD4 T cells plotted against CCR7 and CD3, and CXCR5 and CCR6. Right: Bar graphs showing the frequency of Gag-specific CD154-positive, cytokine-positive cells that express CCR7, CXCR5 and CCR6 (n = 14). *p<0.05.

T_FH_-dependent B cell differentiation requires IL-21. To characterize directly cytokine production from pT_FH_ cells from HIV-uninfected and HIV-infected subjects, we performed intracellular cytokine staining after *ex vivo* SEB stimulation. In addition to surface markers used to define pT_FH_ cells, we detected CD154, IFN-γ, IL-2, IL-17 and IL-21 (Figure 4A). In HIV-uninfected individuals, a minority of CD154-positive, cytokine-positive cells express a CCR7^high^ phenotype (10.1% of IFN-γ positive cells; 28% of IL-2-positive cells; 19.4% of IL-17-positive cells and 17.9% of IL-21-positive cells), while a gradual reduction of cytokine production was found in further differentiated cells based on CXCR5 and CCR6 expression (Figure 4B). However, for all of the cytokines detected, we observed a population of cells that were CCR7^high^CXCR5^high^CCR6^high^, including IL-21-producing cells. Overall, we determined that a mean of 4.5% of CD154-positive IL-21-positive cells were CCR7^high^CXCR5^high^CCR6^high^ (Figure 4B). However, this pT_FH_ population also produced IFN-γ, IL-2 and IL-17 (0.8% of IFN-γ positive cells; 9.0% of IL-2-positive cells and 7.1% of IL-17-positive cells).

Next, we analyzed cytokine production from HIV-infected subjects off-treatment. Overall, we observed a loss of cytokine-producing cells from the CCR7^high^ population and a general shift towards the CXCR5^low^CCR6^low^ population (Figure 4A). Thus, we observed a loss of CCR7^high^CXCR5^high^CCR6^high^ pT_FH_ cells that produce IL-2, IL-17 and IL-21 (Figure 3B; IL-2: 9.0% for HIV-negative vs 2.0% for HIV-positive; IL-17: 7.1% for HIV-negative vs 2.2% for HIV-positive; IL-21: 4.5% for HIV-negative vs 1.1% for HIV-positive).

To analyze HIV-specific cells, PBMC were stimulated with Gag peptide pools and analyzed for cytokine expression. Very few IL-2-positive and IL-17-positive cells were detected within the CM compartment (data not shown). Gag-specific IFN-γ and IL-21-producing cells were detected, however, compared to SEB-stimulation fewer HIV-specific cells expressed CCR7 (4.4% vs 10.7% of IFN-γ positive cells; 3.5% vs 11.9% of IL-21-positive cells for Gag and SEB stimulation, respectively). A majority of HIV-specific cells were not CCR7^high^CXCR5^high^CCR6^high^ (Figure 4C; 0.4% of IFN-γ positive cells and 0.9% of IL-21-positive cells were CCR7^high^CXCR5^high^CCR6^high^).

Overall, we observed IL-21 production from the CCR7^high^CXCR5^high^CCR6^high^ pT_FH_ population, although we detected the most IL-21 in non-pT_FH_ cells, which were CCR7^low^ and CXCR5^low^. In addition to IL-21, the CCR7^high^CXCR5^high^CCR6^high^ pT_FH_ population produced IL-2 and IL-17, cytokines characteristic of T_H1_ and T_H17_ cells, respectively. However, from HIV-infected individuals we observed a loss of CCR7^high^CXCR5^high^CCR6^high^ cells making IL-2, IL-17 and IL-21.

### No relationship between pT_FH_ cells and neutralization activity

**Figure 5:**
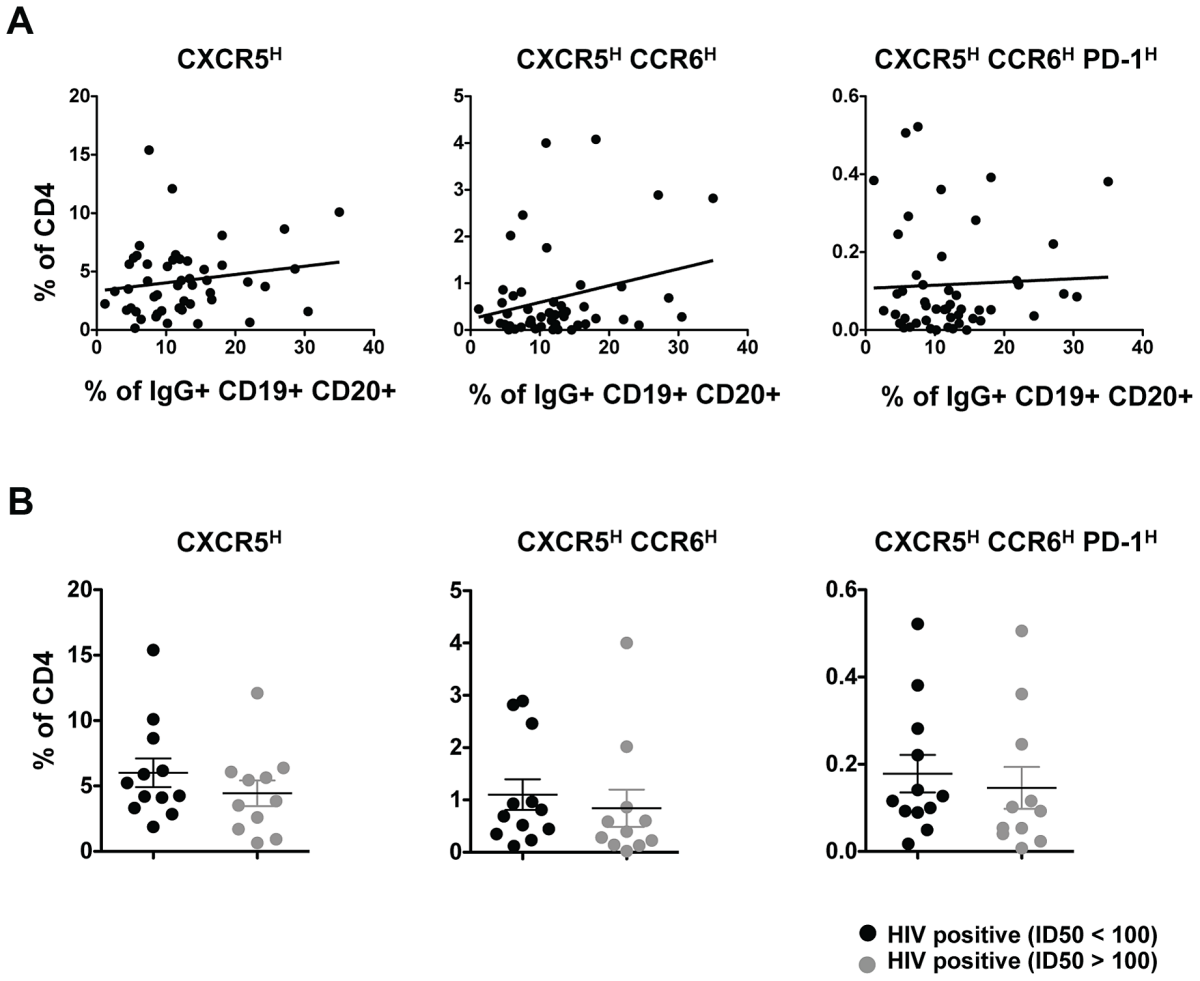
Relationship between pT_FH_ cells and neutralization activity. (**A**) Correlative analysis showing the frequency (%) of CXCR5^high^, CXCR5^high^CCR6^high^ and CXCR5^high^CCR6^high^PD-1^high^ populations in total CD4 cells from HIV-infected (treatment naïve) subjects (n = 50) versus the frequency of IgG+ B cells in the total B population. Correlations were analyzed using the nonparametric Spearman test. (**B**) Pooled data showing the frequency (%) of CXCR5^high^, CXCR5^high^CCR6^high^ and CXCR5^high^CCR6^high^PD-1^high^ populations in total CD4 cells based on neutralization activity (median ID50>100 or <100). No significant differences were determined.

Previous studies have described a relationship between the frequency of peripheral CXCR5^high^ cells and memory B cells and antibody titers with vaccination [16]. Therefore, we analyzed the relationship between the frequency of pT_FH_ cells and IgG-positive memory B cells in PBMC from HIV-infected individuals. We found no significant correlation between the frequency of pT_FH_ cells and IgG-positive B cells (Figure 5A). Similarly, we failed to detect a relationship between the frequency of pT_FH_ and HIV-1 Env-specific antibody titers or total plasma IgG levels (data not shown).

It has also been reported that PD-1^high^ CD4 T cells in blood are associated with cross-clade neutralizing antibody responses during HIV infection [19] and these PD-1^high^ CD4 T cells may represent a population of pT_FH_ cells. Thus, the relationship between pT_FH_ cells and neutralization activity was analyzed using HIV-infected samples classified as good neutralizers (median ID50>100) or poor neutralizers (median ID50<100) [20]. Irrespective of how pT_FH_ cells were defined, we failed to find any relationship between neutralization activity and pT_FH_ cells (Figure 5B).

### Relationship between pT_FH_ cells and T_FH_ cells in human tonsil

**Figure 6:**
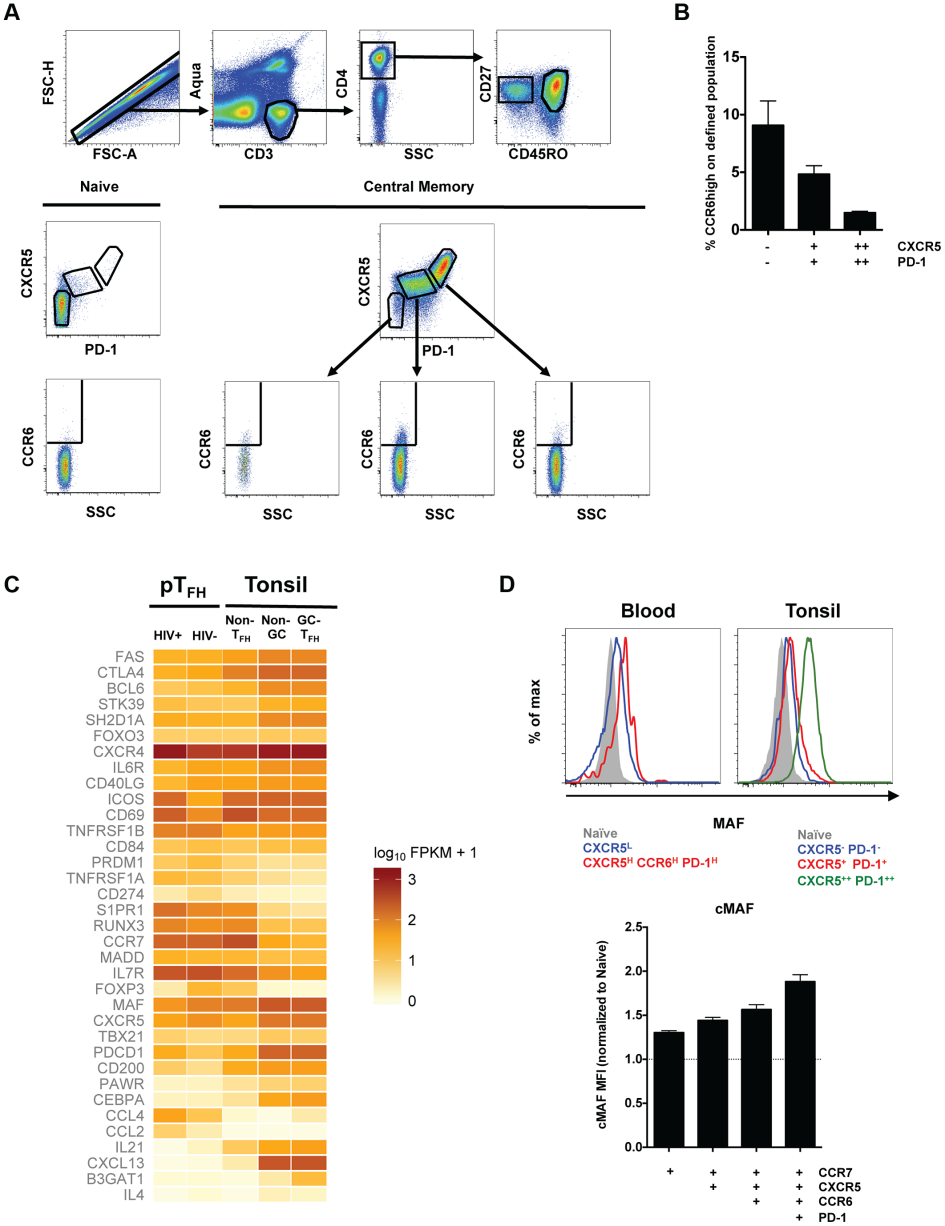
Relationship between pT_FH_ cells and T_FH_ cells in human tonsil. (**A**) Representative flow cytometry plots from HIV-uninfected, pediatric tonsils showing the gating scheme for determining the frequency of CCR6^high^ cells in T_FH_ (CXCR5^high^PD-1^high^) and non-T_FH_ populations. (**B**) Bar graphs showing the frequency of CCR6^high^ cells in T_FH_ and non-T_FH_ populations in human tonsils (n = 5). (**C**) Heatmap analysis of selected genes from RNA-seq data comparing pT_FH_ cells (CXCR5^high^CCR6^high^PD-1^high^) from HIV-uninfected donors, pT_FH_ cells from HIV-infected donors, non-T_FH_ CD4 memory tonsil cells (CM CD57^low^PD-1^dim^CCR7^high^CCR5^low^CXCR4^low^), non-germinal center T_FH_ tonsil cells (CM CD57^low^PD-1^high^CCR7^low^CXCR5^high^) and germinal center T_FH_ tonsil cells (CM PD-1^high^CD57^high^) from HIV-uninfected donors. (**D**) Top: Comparison of MAF expression on CD4 T cells from blood or tonsil. Bottom: Geometric mean (MFI) of MAF expression in the indicated populations of central memory CD4 T cells normalized to MAF MFI in naïve CD4 T cells.

While pT_FH_ cells induce B cell differentiation and immunoglobulin secretion *in vitro*, the relationship between pT_FH_ and T_FH_ cells in secondary lymphoid organs remains unclear. Our *in vitro* coculture studies indicated the greatest isotype-switched immunoglobulin production was elicited from B cells cocultivated with CXCR5^high^CCR6^high^ pT_FH_ cells (Figure 1B). Therefore, we investigated the expression of CCR6 on T_FH_ (CXCR5^high^PD-1^high^) and non-T_FH_ (CXCR5^low^PD-1^low^) tonsil cells to determine if the CXCR5^high^CCR6^high^ pT_FH_ population is related to T_FH_ cells within secondary lymphoid organs (Figure 6A). The lowest frequency of CCR6^high^ cells was found within the CXCR5^high^PD-1^high^ compartment (1.5% of CXCR5^high^PD-1^high^ cells) and the greatest frequency of CCR6^high^ cells within the non-T_FH_ compartment (9% of CXCR5^low^PD-1^low^ cells; Figure 6B).

Similarly, RNA sequence data from the CXCR5^high^CCR6^high^PD-1^high^ pT_FH_ population more closely resembles a memory, non-T_FH_ CD4 T cell population from the tonsil (CM CD57^low^PD-1^dim^CCR7^high^CCR5^low^CXCR4^low^) as compared to the non-germinal center T_FH_ population (CM CD57^low^PD-1^high^CCR7^low^CXCR5^high^) or the GC-T_FH_ population (CM PD-1^high^CD57^high^; Figure 6C). In agreement with previous reports [5], [17], tonsil T_FH_ populations expressed higher levels of BCL6, IL-21, and CXCL13, and lower levels of PRDM1 and S1PR1 compared to the non-T_FH_ memory population. The pT_FH_ population from HIV-uninfected individuals expressed comparable levels of S1PR1 and PRDM1 to the non-T_FH_ memory population in the tonsil (Figure 6). We also observed lower transcript levels of MAF, BCL6, IL-21, and CXCL-13 in the pT_FH_ population compared to tonsillar T_FH_ populations. Importantly, MAF protein expression was highest in the CCR6^high^PD-1^high^ pT_FH_ population compared to other peripheral populations, although still lower than tonsillar T_FH_ cells. (Figure 6D). For many of the selected genes, pT_FH_ cells from HIV-infected subjects were comparable to pT_FH_ from HIV-uninfected individuals, however, we observed greater transcript levels of activation molecules such as ICOS and CD69. Additionally, the levels of IL-21 were decreased in pT_FH_ cells from HIV-infected individuals, supporting earlier results (Figure 4B). Collectively, our data suggest the pT_FH_ population characterized as CXCR5^high^CCR6^high^ most closely resembles a non-T_FH_ memory population in the tonsil.

## Discussion

The development and nature of human T_FH_ memory cells following an effector immune response are not known. The ability to define a population of memory T_FH_ cells in PBMC (pT_FH_) would help inform our understanding of CD4 T cell dynamics within lymphoid tissue during vaccination or infection. Studies of chronic infection may be helpful in this regard [21]. Whether the accumulation of T_FH_ cells during chronic infection [4], [15] impacts the T_FH_ memory population is of particular interest, especially if memory T_FH_ cells migrate between lymphoid organs and peripheral tissues. Recent studies [14], [16] have suggested that circulating CXCR5^high^ CD4 T cells may represent the peripheral counterparts of T_FH_ cells. However, the relationship between pT_FH_ and T_FH_ cells within secondary lymphoid organs remains unclear. Therefore, it is of great relevance to determine if pT_FH_ cells originate from GC-T_FH_ cells and represent a memory T_FH_ population, reflect a precursor population that differentiates into GC-T_FH_ upon re-exposure to antigen, or both. Our studies begin to address these issues by further defining pT_FH_ cells, comparing pT_FH_ cells to tonsillar T_FH_ cells, and analyzing the effect of HIV on these cells.

In concordance with previous studies, we showed that circulating CXCR5^high^ CD4 T cells support B cell differentiation *in vitro* [14], [17]. A majority of the CXCR5^high^ cells expressed CD150, and while CD150 was used for gating in the co-culture assays, we found it did not impact the loss of pT_FH_ cells or effect our results with respect to loss of pT_FH_ cells, recovery with ART or lack of association with B cell or antibody responses (data not shown). However, within the CXCR5^high^ population the expression of CCR6 and PD-1 did further define pT_FH_ populations with differential abilities for naïve B cell help and isotype switching. Thus, pT_FH_ cell populations support both the activation and maturation of naïve B cells, and immunoglobulin isotype switching. Correspondingly, the individual pT_FH_ populations produced cytokines associated with B cell maturation and survival, such as IL-21 [22], IL-2 [23] and IL-17 [24], in contrast to T_FH_ cells within secondary lymphoid tissue, which display a limited cytokine profile that includes IL-4, IL-10 and IL-21, but compromised production of IL-2 and IL-17 [4]. Whether these pT_FH_ populations represent different stages of T_FH_ memory development or originate from separate CD4 T cell populations within lymphoid tissue [25] is still unclear.

In order to better understand the relationship between T_FH_ and pT_FH_ cells, we compared gene expression levels between pT_FH_ and tonsillar CD4 T cell populations and focused on genes important for T_FH_ differentiation, migration, and function. We found that the pT_FH_ population with the greatest B cell helper function most closely resembled a CM, non-T_FH_ CD4 T cell subset within the tonsil. While our studies do not directly address the relationship between GC-T_FH_ in lymph nodes and circulating CD4 T cells from the same patients, our data challenge whether pT_FH_ are memory T_FH_ cells. A recent study reported that germinal center T_FH_ cells in mice migrate throughout the follicle, but generally do not leave the follicle to enter the blood [26]. While it is conceivable that pT_FH_ cells represent a very minor population of T_FH_ cells that exit the follicle, it is also possible that pT_FH_ cells are reflective of a precursor T_FH_ population that exits the lymphoid organ and enters the circulation before entering the follicle. However, while we find the CXCR5^high^CCR6^high^PD-1^high^ pT_FH_ population does not resemble a memory T_FH_ population, Locci and colleagues found a CXCR5+CXCR3-PD-1+ pT_FH_ subset that functionally and transcriptionally resembles a memory T_FH_ population [27]. A recent study in mice reported that memory T_FH_ cells have reduced mRNA expression of T_FH_ markers such as Bcl6, IL-21, ICOS and PD-1 compared to the effector T_FH_ population [28], indicating the expression of these molecules may change depending on the phase of infection. Therefore, further investigation of pT_FH_ subsets and their relationship to memory and effector populations at multiple stages of infection is needed.

pT_FH_ and naïve B cell co-cultures from HIV-infected subjects produced fewer immunoglobulins compared to co-cultures from HIV-uninfected subjects. The observed defect in immunoglobulin production is likely due to impaired pT_FH_ help to B cells instead of B cell dysfunction, as co-cultures included naïve B cells rather than memory B cells that exhibit abnormalities in HIV infection [29]. Furthermore, while co-culture supernatants from HIV-infected subjects demonstrated a heterogeneous cytokine profile, similar to HIV-uninfected subjects, intracellular cytokine staining showed that fewer CCR7^high^CXCR5^high^CCR6^high^ pT_FH_ cells produced IL-2, IL-17 and IL-21 in chronic HIV infection compared to HIV-uninfected individuals. Furthermore, gene expression analysis of HIV-infected pT_FH_ revealed fewer IL-21 and IL-4 transcripts, although the overall levels of cytokine transcripts were low.

Recent studies have shown T_FH_ cells within secondary lymphoid organs accumulate in some donors or animals during chronic HIV/SIV infection and that T_FH_ accumulation is associated with GC B cell expansion and increased serum immunoglobulin concentrations [4], [22], [30]. In contrast to T_FH_ cells, our studies revealed pT_FH_ cells consistently decrease in chronic HIV infection, with disease progression resulting in a greater reduction of these compartments within the total CD4 T cell population. However, it should be noted that we were unable to analyze T_FH_ cells within secondary lymphoid organs from these subjects and therefore we are unable to directly compare the frequency of pT_FH_ cells and T_FH_ cells from the same individual. The differences between the increase in T_FH_ cells and decrease in pT_FH_ cells may be due to differences in disease state (i.e. early vs late infection) or represent a steady state of T_FH_ cells trafficking between the lymphoid tissue and the blood. The decreased frequency of pT_FH_ in the blood may indicate impaired ability of T_FH_ to exit the lymph node in chronic HIV infection where the tissue architecture is not intact. Alternatively, the decreased frequency of pT_FH_ in the blood may be a result of pT_FH_ trafficking to secondary lymphoid organs. In agreement with previous studies [14], [17], we found a majority of CXCR5^high^ cells express CCR7, and it has previously been suggested that pT_FH_ cells migrate to secondary lymphoid organs upon infection due their expression of CCR7 and CD62L [14].

A confounding factor with regard to how we interpret the decrease in pT_FH_ cells is that we also found a reduction in the surface expression of CXCR5 on CD4 T cells in chronic HIV infection, which may result from increased sera levels of CXCL-13 [31], [32]. Furthermore, our co-culture data indicate that CXCR5^low^ CD4 T cells from viremic subjects can induce some B cell differentiation. These data support the possibility that in chronic HIV infection, a subset of functional pT_FH_ cells may be phenotypically defined as CXCR5^low^. Additionally, it should be noted that analysis of cellular subsets within the CXCR5^high^ population in chronic HIV infection revealed the frequency of CCR6^high^PD-1^high^ cells increased. These results are consistent with a state of generalized immune activation, as we also observed increased surface expression of ICOS on CXCR5^high^ and CXCR5^high^PD-1^high^ cells, and a positive association between the frequency of PD-1^high^ cells within the CXCR5^high^ population and serum concentrations of soluble CD14 [33]. Similarly, gene expression analysis indicated increased transcript levels of activation markers, such as ICOS and CD69 within the pT_FH_ population during HIV infection. Overall, these data emphasize the difficulty in defining pT_FH_ cells in chronic HIV infection and understanding the relationship between pT_FH_ cells and T_FH_ cells.

The uncertain definition of pT_FH_ cells in HIV infection may provide an explanation as to why we were unable to identify correlations between pT_FH_ populations and circulating IgG-positive memory B cells, or between pT_FH_ cells and HIV-specific IgG (data not shown). Furthermore, we found no correlation between the frequency of pT_FH_ and the neutralization activity of a well-characterized cohort of HIV-infected donors [20]. However, the absence of a correlation between pT_FH_ cells and circulating HIV Env-specific IgG may also be explained by the lack of a time-dependent association (early vs. late infection) between T_FH_ and pT_FH_ cells, or indicate that the generation of IgG and broadly neutralizing antibodies is regulated by parameters other than pT_FH_, confounded by T-cell independent antibody production commonly observed in HIV infection [34] or generalized immune activation. Thus, our data challenge the application of the pT_FH_ population as a surrogate of GC T_FH_-B cell interactions in chronic HIV infection. While our studies did not find a correlation between pT_FH_ cells and neutralizing antibodies, several recent studies, each with a different definition of pT_FH_ cells, have reported an association with antibody responses during vaccination, infection or autoimmune disease [27], [35]–[37]. Therefore, further studies are needed to establish the association between pT_FH_ subsets and the generation of neutralizing antibodies, especially in HIV infection.

Overall, our data indicate that a range of circulating CD4 T cell populations can provide B cell help, possibly through differential secretion of soluble factors and/or cell-cell contact interactions [17], [35] and that HIV infection results in loss of these cells over time, but with relative increases within the CXCR5^high^ compartment which may be explained by immune activation. Furthermore, we did not find any association between pT_FH_ and measures of B cell function such as HIV neutralization breadth/potency, HIV-specific IgG, or total IgG, suggesting application of this population as a surrogate of GC T_FH_-B cell interactions during HIV infection may be limited. A better understanding of the differentiation process and the developmental relationship between pT_FH_ subsets and lymph node T_FH_ cells is critical for the establishment of reliable peripheral blood CD4 T cell correlates for monitoring infection- or vaccine-associated B cell responses.

## Materials and Methods

### Ethics statement

Signed informed consent was obtained in accordance with the Declaration of Helsinki and approved by the appropriate Institutional Review Board. Tonsil cells were acquired from anonymized discarded pathologic specimens from Children's National Medical Center (CNMC) under the auspices of the Basic Science Core of the District of Columbia Developmental Center for AIDS Research. The CNMC Institutional Review Board determined that study of anonymized discarded tonsils did not constitute ‘human subjects research.’

### Subjects

Fresh HIV-uninfected peripheral blood mononuclear cells (PBMC) were obtained from individuals participating in the NIH research apheresis program. Fresh HIV-infected blood was obtained from the Vaccine Research Center Clinic or Drexel University College of Medicine. Frozen HIV-infected PBMC were obtained from three study populations (Table S1). For untreated HIV infection, cells were obtained from volunteers who participated in a therapeutic vaccination trial (no efficacy was observed) conducted in the 1990's prior to the advent of combination antiretroviral therapy (cART) [38]. The second study population consisted of donors from a cohort used to identify individuals with HIV broadly neutralizing antibodies [20]. To study the effect of cART, we obtained PBMC from HIV-infected donors participating in AIDS Clinical Trials Group study A5142 prior to initiation of cART and 24 and 48 weeks post-therapy [39], [40]. PBMC and tonsil cells were cultured in RPMI 1640 supplemented with 10% fetal bovine serum, 2 mM L-glutamine, 100 U/mL penicillin and 100 µg/mL streptomycin (Invitrogen).

### Antibodies

Directly conjugated antibodies were acquired from the following: (1) BD Biosciences: CD3-H7APC, CXCR5-Alexa488 (RF8B2), CCR7-Alexa700, IgG-APC, IFN-γ-Alexa700 and IL-21-Alexa647 (3A3-N2.1) (2) Beckman Coulter: CD45RO-ECD and CD27-PC5 (3) Biolegend: CCR7-BV421, CCR6-PE (TG7/CCR6), CCR6-Alexa647 (TG7/CCR6), CD20-BV570, CD150-PE, IL-2-BV605, IL-17a-Cy5.5PerCP and CD154-Cy5PE (4) Invitrogen: CD4-Cy5.5PE, CD27-QD655, CD27-QD605 and CD19-PacBlue (5) Southern Biotech: IgD-FITC and IgD-PE (6) eBioscience: cmaf-eFluor660 (sym0F1), CXCR5-PerCP-efluor710 (MU5UBEE). Biotinylated anti-PD-1 was from R&D and streptavidin-Cy7PE (or QD655) was from Molecular Probes. The following antibodies were conjugated in our lab: CD19-QD705 and CD57-QD565. Quantum dots and Aqua amine viability dye were obtained from Invitrogen.

### Polychromatic flow cytometry

#### Phenotypic analysis

1–2×10^6^ PBMC were incubated with Aqua-dye and surface stained with titrated amounts of anti-CD3, anti-CD4, anti-CD27, anti-CD45RO, anti-CCR7, anti-CXCR5, anti-CD150, anti-CCR6, anti-PD-1 and anti-CD19. Post-wash, cells were incubated with fluorescent-conjugated streptavidin, washed and fixed with 1% paraformaldehyde. 

#### Intracellular cytokine staining

3×10^6^ PBMC were incubated in 1 mL of medium containing brefeldin A (10 ug/mL) in the absence or presence of HIV-1 Gag-peptide pools (15mers overlapping by 11 residues; NIH AIDS Research and Reference Reagent Program) or 1 ug/mL SEB (Sigma) for 6 hours. Cells were surface stained, permeabilized (Cytofix/Cytoperm kit; BD Biosciences), and stained with anti-CD3, anti-IFN-γ, anti-IL-2, anti-IL-17a, anti-IL-21 and anti-CD154. Events were collected on a modified LSRII flow cytometer (BD Immunocytometry Systems) and electronic compensation was performed with antibody capture beads (BD Biosciences). Data were analyzed using FlowJo Version 9.6 (TreeStar).

### T and B cell culture

Co-culture experiments were performed with freshly isolated PBMC. 5×10^4^ CD4 T cell populations were sorted based on expression of CCR7, CXCR5, CD150, CCR6 and PD-1 and cultured with 5×10^4^ autologous naïve B cells (1∶1 ratio) in the presence of SEB (0.5 µg/ml). Supernatants harvested on Day 2 were analyzed for cytokines using Luminex technology (Milliplex MAP Kit, HTH17MAG-14K, Millipore). The lower limit of detection (LOD) was set at the lowest concentration on the standard curve and values below the LOD were counted as zero. Supernatants collected on Day 12 were analyzed for immunoglobulins (Milliplex MAP Kit, HGAMMAG-301K). Some supernatants exceeded the saturation limit of the standard curves for IgM and IgG3. These values were included in the analysis and quantified as being equivalent to the highest determined concentration.

### ELISA

Soluble CD14 and CXCL-13 (R&D Systems) were measured in plasma or sera from HIV-infection patients according to the manufacturer's instructions.

### CXCL-13 treatment

Freshly isolated PBMCs were incubated with recombinant human CXCL-13 (R&D Systems) at 37°C or 4°C and analyzed for CXCR5 surface expression by FACS.

### Illumina deep sequencing of messenger RNA

CD4 T cell populations were sorted from uninfected PBMC (n = 5), HIV-infected PBMC (n = 5) and uninfected human tonsils (n = 4) based on expression of CCR7, CXCR5, CD150, CCR6 and PD-1 for PBMC and CD57, PD-1, CCR7, CXCR5, CCR5 and CXCR4 for tonsils. Total RNA was purified from sorted cell populations and treated with DNAse I (Ambion) to minimize genomic DNA contamination. Polyadenylated RNA was isolated using Oligo-dT Dynabeads (Life Technologies), chemically fragmented, and used to construct barcoded Illumina Truseq libraries. Libraries were size-selected, quantified, pooled, size-selected and quantified again, and clustered on an Illumina Truseq Paired-End Flowcell v3. The flowcell was sequenced on an Illumina HiSeq 2000 in a 2×75-base paired-end, indexed run. Adaptor sequence was trimmed from the raw sequencing reads using Trimmomatic. The trimmed sequencing reads were subsequently aligned to the human genome (hg19) using TopHat 2. Differential expression testing was done using Cufflinks 2 and visualization of differential expression was done using the R package cummerbund. Accession numbers of the selected genes are shown in Supporting Table S2.

### Virus neutralization

Neutralization activity of patient sera was determined against 20 viral isolates using a TZM-bl neutralization assay as previously described [20].

### In vitro infection

Freshly isolated PBMCs were stimulated with PHA (10 µg/ml). After 12 hours stimulation, CXCR5^high^ cells were sorted by FACS Aria based on surface molecule expression and infected by a multiplicity of infection (MOI) of 0.01 with either HIV NL-E or HIV NLAD8-E [41]. The infected cells were cultured in the presence of 50 U/ml recombinant human interleukin-2 (R&D) for 5 days and analyzed for CXCR5 expression by FACS.

### Statistics

Experimental variables were analyzed using the nonparametric Mann-Whitney U test, the Wilcoxon matched-pairs signed rank test or the Friedman test with Dunn's multiple comparison post-test. Correlation analysis was performed using the nonparametric Spearman test. Error bars depict mean+SEM in all bar graphs shown. The GraphPad Prism statistical analysis program (GraphPad Software, version 5.0) was used throughout.

## Supporting Information

Figure S1 **Characterization of peripheral T_FH_ cells.** (**A**) Scatter plot depicting frequency of IgG+ and IgA+ B cells in total B (CD19+) or naïve B cells (CD19^+^CD27^low^IgD^high^) for HIV-uninfected (n = 5), HIV-infected (non-viremic; n = 7) and HIV-infected (viremic; n = 1) donors. Both surface and intracellular staining of IgA and IgG were used to determine frequency. (**B**) Left: Representative flow cytometry plots from HIV-uninfected PBMC showing CXCR3 and CCR6 expression within CXCR5^high^ and CXCR5^low^ CD4 T cell subsets. Right: Scatter plot comparing the frequency of CXCR3 and CCR6 subsets within the CXCR5^high^ population from HIV-uninfected (open circles; n = 4) and HIV-infected individuals (closed circles; n = 8).
(EPS)

Figure S2 **Decrease of pT_FH_ cells in HIV infection.** (**A**) Pooled data showing the frequency (%) of indicated populations in CXCR5-expressing cells from PBMC from HIV-uninfected (open circles; n = 13), HIV-infected (treatment-naïve), CD4 count >200 (light gray circles; *n* = 44), and HIV-infected (treatment-naïve), CD4 count <200 (black circles; *n* = 22). Significant differences between uninfected and HIV-infected subjects were determined using the Mann-Whitney U test. ***p<0.001; **p<0.01; *p<0.05. Far Right: Correlative analysis between the frequency of CXCR5^high^PD-1^high^ cells and the concentration of soluble CD14 in the sera or plasma. The trend did not reach statistical significance as determined by the Spearman test. (**B**) Top: Longitudinal analysis showing the frequency (%) of indicated populations in CXCR5-expressing cells from HIV-infected (treatment naïve) subjects (n = 10) over 36–48 months. No significant correlations were found. Bottom: Longitudinal analysis showing CD4 counts and viral loads from HIV-infected (treatment naïve) subjects (n = 10) over 36–48 months. (**C**) Pooled data showing the frequency (%) of indicated populations in CXCR5-expressing cells from PBMC from HIV-uninfected subjects (open circles; n = 13) and HIV-infected subjects before (n = 14, week 0; black circles) and after ART (week 24, dark gray circles; week 48, light gray circles).
(EPS)

Figure S3 **Characterization of pT_FH_ cells in HIV infection.** (**A**) CCR7^high^CXCR5^low^ and CCR7^high^CXCR5^high^CCR6^high^ CD4 T cells isolated from PBMCs were cultured with autologous naïve B cells (CD19^high^CD27^low^IgD^−^) in the presence of SEB for 2 days and cytokine concentrations were measured from supernatants (HIV-uninfected, n = 5; HIV-infected (non-viremic), n = 4, HIV-infected (viremic), n = 0–1). Due to limited cell numbers we were unable to collect CCR7^high^CXCR5^high^CCR6^high^ cells from viremic individuals. (**B**) Sorted CXCR5^high^ central memory cells isolated from blood do not down-regulate surface expression of CXCR5 upon X4 or R5 *in vitro* infection.
(EPS)

Table S1 **CD4 count, viral load and neutralization activity of subjects studied.**
(EPS)

Table S2 **Accession numbers of selected genes.**
(EPS)
